# Supplementary material for: Recovery of neuropsychological function following abstinence from alcohol in adults diagnosed with an alcohol use disorder: Protocol for a systematic review of longitudinal studies
Source: PLoS One. 2022 Sep 29;17(9):e0274752. doi: 10.1371/journal.pone.0274752 (PMC9521940; doi:10.1371/journal.pone.0274752)
Supplement: S1 File — (PDF) [file pone.0274752.s002.pdf]

Recovery of neuropsychological function following abstinence from alcohol in adults diagnosed with an Alcohol Use Disorder: Protocol for a systematic review of longitudinal studies

Anna Powell, Harry Sumnall, Jessica Smith, Rebecca Kuiper, Catharine Montgomery

S1 File.

Systematic search strategies for APA PsycInfo, EBSCO MEDLINE, CINAHL, and Web of Science

Source(s)	Search Strategy
**APA PsycInfo**, accessed via https://www.proquest.com/	i. #1 ab(alcoholism OR alcoholic*)<br>ii. #2 ab(alcohol OR drinking) AND ab(abus* OR addict* OR dependen* OR disorder* OR harmful)<br>iii. #3 #1 OR #2<br>iv. #4 ab(recover* OR abstinen* OR sober OR treatment)<br>v. #5 #3 AND #4<br>vi. #6 (cogniti* OR neuropsycholog*OR executive)<br>vii. #7 ti(learning OR attention OR orientation OR switching OR shifting OR updating OR flexibility OR initiating OR motor OR planning OR “problem solving” OR “functional ability” OR “decision making” OR “time manag*” OR inhibit* OR monitor* OR “goal directed” OR “mental process*” OR memory OR dysexecutive OR intelligence OR IQ OR gait OR posture OR balance OR propriocept* OR “emotional function*” OR “emotion recognition” OR “emotional processing” OR language OR sensory OR perception OR vibrotactile OR visuospatial OR spatial OR "reaction time" OR "processing speed" OR "temporal order judgement" OR "amplitude discrimination" OR "duration discrimination" OR coordination)<br>viii. #8 #6 OR #7<br>ix. #9 #5 AND #8<br>x. #10 ab(cohort OR prospective OR longitudinal OR “follow up*” OR retrospective OR “repeated measures” or timepoin* or “time poin*”))<br>xi. #11 #9 AND #10<br>xii. Filters – 2000-2022
**MEDLINE (EBSCO)** and **CINAHL**, accessed separately via https://web.a.ebscohost.com	i. S1 (MH “Alcohol-Related Disorders+”)<br>ii. S2 (MH “Alcoholism”)<br>iii. S3 AB (alcohol OR drinking)<br>iv. S4 AB (abus* OR addict* OR dependen* OR disorder* OR harmful)<br>v. S5 S3 AND S4<br>vi. S6 S1 OR S2 OR S5<br>vii. S7 AB recover* OR abstinen* OR sober OR treatment<br>viii. S8 S6 AND S7<br>ix. S9 (MH “Cognition Disorders+”)

	x. S10 (MH “Cognition+”)<br>xiii. S11 TX (cogniti* OR neuropsycholog*OR executive)<br>xi. S12 TI learning OR attention OR orientation OR switching OR shifting OR updating OR flexibility OR initiating OR motor OR planning OR “problem solving” OR “functional ability” OR “decision making” OR “time manag*” OR inhibit* OR monitor* OR “goal directed” OR “mental process*” OR memory OR dysexecutive OR intelligence OR IQ OR gait OR posture OR balance OR propriocept* OR “emotional function*” OR “emotion recognition” OR “emotional processing” OR language OR sensory OR perception OR vibrotactile OR visuospatial OR spatial OR "reaction time" OR "processing speed" OR "temporal order judgement" OR "amplitude discrimination" OR "duration discrimination" OR coordination<br>xii. S13 (MH “Task Performance and Analysis+”)<br>xiii. S14 (MH “Psychological Tests+”)<br>xiv. S15 S9 OR S10 OR S11 OR S12 OR S13 OR S14<br>xv. S16 S8 AND S15<br>xvi. S17 AB cohort OR prospective OR longitudinal OR “follow up*” OR retrospective OR “repeated measures” OR 2timepoint* OR “time poin*”<br>xvii. S18 S16 AND S17<br>xviii. Filters – 2000-2022, middle aged: 45-64 years, adult: 19-44 years, adult: 19+ years, adolescent: 13-18 years, young adult: 19-24 years
Web of Science Core Collection; accessed via https://www.webofscience.com/wos/	i. #1 AB=(alcoholism OR alcoholic*)<br>ii. #2 AB=(alcohol OR drinking)<br>iii. #3 AB=(abus* OR addict* OR dependen* OR disorder* OR harmful)<br>iv. #4 #2 AND #3<br>v. #5 #1 OR #4<br>vi. #6 AB=(recover* OR abstinen* OR sober OR treatment)<br>vii. #7 #5 AND #6<br>viii. #8 ALL=(cogniti* OR neuropsycholog*OR executive)<br>ix. #9 TI=(learning OR attention OR orientation OR switching OR shifting OR updating OR flexibility OR initiating OR motor OR planning OR “problem solving” OR “functional ability” OR “decision making” OR “time manag*” OR inhibit* OR monitor* OR “goal directed” OR “mental process*” OR memory OR dysexecutive OR intelligence OR IQ OR gait OR posture OR balance OR propriocept* OR “emotional function*” OR “emotion recognition” OR “emotional processing” OR language OR sensory OR perception OR vibrotactile OR visuospatial OR spatial OR "reaction time" OR "processing speed" OR "temporal order judgement" OR "amplitude discrimination" OR "duration discrimination" OR coordination)<br>x. #10 #8 OR #9<br>xi. #11 #7 AND #10<br>xii. #12 AB=(cohort OR prospective OR longitudinal OR “follow up*” OR retrospective OR “repeated measures” or timepoin* “time poin*”)<br>xiii. #13 #10 AND #12<br>xiv. Filters – 2022 OR 2021 OR 2020 OR 2019 OR 2018 OR 2017 OR 2016 OR 2015 OR 2014 OR 2012 OR 2011 OR 2009 OR 2010 OR 2008 OR 2007 OR 2006 OR 2005 OR 2004 OR 2003 OR 2002 OR 2001 OR 2000
