# Supplementary material for: Recovery of neuropsychological function following abstinence from alcohol in adults diagnosed with an alcohol use disorder: Protocol for a systematic review of longitudinal studies
Source: PLoS One. 2022 Sep 29;17(9):e0274752. doi: 10.1371/journal.pone.0274752 (PMC9521940; doi:10.1371/journal.pone.0274752)
Supplement: S2 File — (PDF) [file pone.0274752.s003.pdf]

Recovery of neuropsychological function following abstinence from alcohol in adults diagnosed with an Alcohol Use Disorder: Protocol for a systematic review of longitudinal studies

Anna Powell, Harry Sumnall, Jessica Smith, Rebecca Kuiper, Catharine Montgomery

S2 File.

Data extraction form

Note: where information is not available, highlight form description (e.g., age range) for clarity in deciding if correspondence with author required.

**1. General Information**	
Record title<br>*First few characters of primary study author's name*	
Person extracting	
Date<br>*Of study publication*	
Study Title	
Authors<br>*Including lead author contact details*	
Study funding source	
Possible conflicts of interest	

**2. Study method/characteristics**	
Design<br>*Cohort – prospective or retrospective*	
Setting<br>*May refer to hospital/community, inpatient/outpatient, rural/urban etc.*	
Location<br>*Country and region*<br>*AP to check WHO region and related economic status at the time of study*	
Participants<br>*Sample size (and how calculated/justified), age (range; mean), gender (male n, %; female n, %), sample size (at each time point), any other relevant characteristics*	
Exposure(s)<br>*Exact alcohol diagnosis and tool used:*<br>*DSM-5 AUD (mild/moderate/severe)*<br>*DSM-4 alcohol dependence vs abuse*<br>*ICD-10/11 dependence vs harmful use.*	

If available - length of diagnosis, no. treatment attempts, age of first drink, details of alcohol use (type/frequency/intensity/duration), and length of abstinence at each time point	
Co-morbidities<br>*Study cannot be defined by co-morbidities (e.g., all patients have both AUD and ADHD), but if any co-morbidities are reported in the sample, state these*	
Other reported substance use<br>*Again, study cannot be defined by this, but note if any reported in the sample, and whether current or past*	
Comparison group<br>*Are these non-AUD controls, individuals with different AUD severity, or with different lengths of abstinence?*<br>*Age, gender, country/location, sample size (at each time point), diagnosis, other relevant characteristics, how many times tested, how chosen, were they matched?*	
Recruitment procedures<br>*Including inclusion/exclusion criteria*	
Details of administration<br>*Any details on the duration of the study, timeline of assessment (initial assessment, follow-ups), follow-up methods and any other details of them. Specify whether each follow-up before, during, or after active AUD, and any relapse details.*	
Details of participants leaving study at each time-point<br>*Characteristics of those who left via attrition, or exclusion by the research team/clinical staff (and details given)*	

**3. <u>Primary Outcome (dependent variable)</u>**	
Neuropsychological function measure(s) used<br>*At each time point, name the measure (e.g., validated questionnaires such as BRIEF-A, EFI, validated tests such as n-back, or medical diagnosis of cognitive impairment)*<br>*If uncertain about validation status - name measure and highlight in yellow for discussion.*	
Outcome findings at each time-point<br>*Describe findings*<br>*Give context (e.g., is score good/bad)*	

Measures of dispersion (name, e.g., M/SD, range/interquartile range) and give values<br>*State if values adjusted in relevant analysis for covariates/confounders.*	
Effect sizes for each outcome result<br>*Measure of effect size (type, value, and if available, context such as small/ moderate/ large)*<br>*Specify if adjusted for covariates/confounds.*	
P values for each outcome result<br>*To as many decimal places as available*<br>*Specify if adjusted for confounds/covariates*	
Confidence intervals for each outcome result<br>*To as many decimal places as available*<br>*Specify if adjusted for confounds/covariates*	
Statistical analyses/technique<br>*E.g., regression, analysis of variance etc.*<br>*Note if raw scores standardised (useful to compare performance on different tests)*<br>*Was attrition accounted for to reduce bias? E.g., regression imputation, weighting, multiple imputation. Or statistically ignored (complete subject analysis or description of characteristics)*	
Details of control/adjustment for confounding factors<br>*Experimental methods - randomisation, restriction, or matching*<br>*Analytical methods - stratification, or statistical modelling (Multiple or conditional Logistic Regression, Linear Regression, Cox proportional hazards regression, multivariable regression analysis, Analysis of Covariance). If analytical, note how confounds measured*	

**4. Secondary Aims**	
Did paper assess predictors of neuropsychological function recovery?	
If so what, and how measured/classified?<br>*Some possible predictors might be AUD severity/duration, treatment*	

attempts/adherence, age of first drink, mood disorders etc.	
What were the findings?<br>*Description for result at each available time point, effect sizes, p values, confidence intervals, statistical techniques used, describe adjustments for confounding factors and attrition.*	

**5. Quality Assessment**	
JBI Cohort Study Checklist score	

**6. Extra Information**	
Does the study directly address the review objective?	
Reviewer comments<br>*Any extra details. If uncertain about any elements and want discussion, mention here, and highlight*	
Is correspondence needed for further study information?<br>*What and from whom?*<br>*Give date requested*	
Correspondence received?<br>*What/when/from whom*<br>*If not received within a month, and the details are key, exclude.*	
